# Renal tuberculosis mimicking renal cell carcinoma: a case report

**DOI:** 10.1186/s13256-019-2073-0

**Published:** 2019-05-11

**Authors:** Kays Chaker, Marouene Chakroun, Maroua Gharbi, Mohamed Chebil

**Affiliations:** Departement of Urology, Charles Nicolle Hospital, Tunis, Tunisia

**Keywords:** Urogenital tuberculosis, Pseudotumor, Renal cell carcinoma

## Abstract

**Background:**

Urogenital tuberculosis is still a frequent presentation, and it constitutes a current public health problem in endemic areas. The clinical presentation of this form of the disease may be misleading. The pseudotumoral type of renal tuberculosis is extremely uncommon.

**Case presentation:**

We present a case of a 52-year-old African woman who presented with urogenital tuberculosis in its pseudotumoral form. This case was initially diagnosed and managed as renal cancer. Histopathology confirmed the diagnosis of pseudotumoral renal tuberculosis.

**Conclusions:**

The pseudotumoral form of urinary tuberculosis can be difficult to diagnose. Only bacteriological or histological confirmation allows diagnosis for adequate treatment.

## Background

Urogenital tuberculosis is a common presentation of extrapulmonary tuberculosis, with kidneys being the most commonly involved site. Renal tuberculosis usually presents with nonspecific symptoms such as pyuria, dysuria, fever, weight loss, and flank pain. Renal tuberculosis can be revealed by a mass, usually due to hydronephrosis of the involved kidney. Pseudotumoral presentation of renal tuberculosis is an extremely rare entity [1].

## Case presentation

**Fig. 1:**
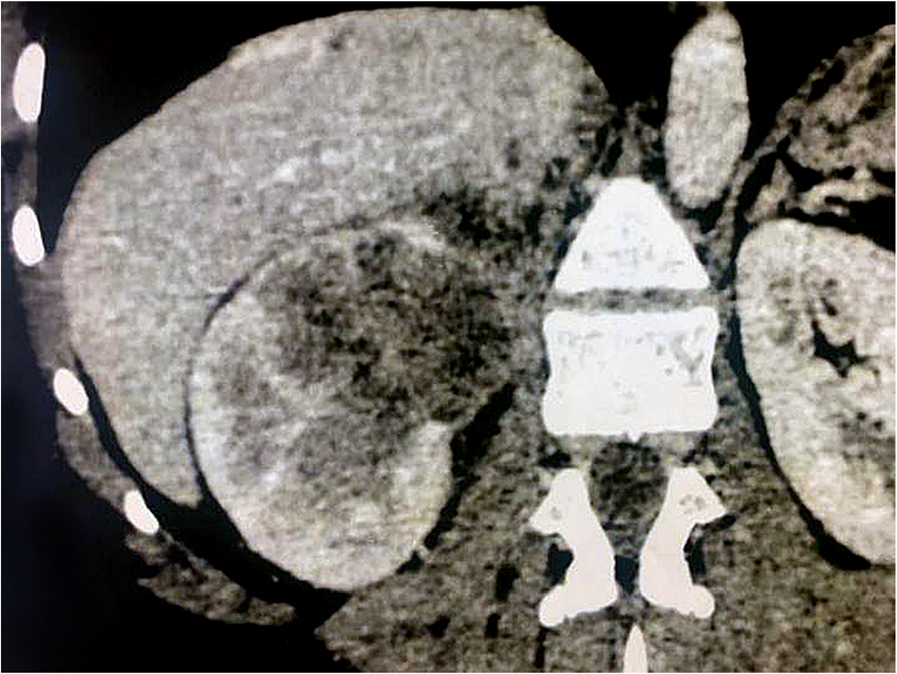
Computed tomography shows heterogeneous enhancing mass involving the upper and middle poles of the right kidney measuring approximately 8 × 7 cm

A 52-year-old African woman presented to our department complaining of 8 months of fever with hematuria, weight loss, decreased appetite, generalized weakness, and intermittent right flank pain. She had a history of pulmonary tuberculosis treated for a 6-month period 10 years ago. Her physical examination was unremarkable. Her temperature was 37.7 °C, blood pressure 124/84 mmHg, and pulse rate regular at 86 beats/min. Laboratory investigations revealed hemoglobin of 10 g/dl, total leukocyte count 15,000/mm^3^, and elevated erythrocyte sedimentation rate of 150 mm/hr. Liver function test and other biological investigation results were normal. Urinalysis demonstrated urinary pH 6.0, leukocytes 1+, protein 4+, erythrocytes 3+, uncountable leukocyte casts, and negative culture of the urine for pyogenic agents. Abdominal color Doppler ultrasound revealed an enlarged right kidney measuring approximately 8 × 6 cm with minimal flow. Contrast-enhanced computed tomography of the abdomen subsequently revealed a large heterogeneously enhancing mass in the right kidney, measuring approximately 8 × 7 cm, giving a radiological impression of renal cell carcinoma (Fig. 1). An enhanced computed tomographic scan showed a normal bladder. No hydronephrosis or wall thickening of the ureter was seen. Considering the clinical presentation as well as laboratory and radiological investigations, a provisional diagnosis of renal cell carcinoma was made, and the patient underwent an open right radical nephrectomy using a transperitoneal approach in view of the large size of the lesion. Radical nephrectomy of the specimen was sent for histopathological examination.

**Fig. 2:**
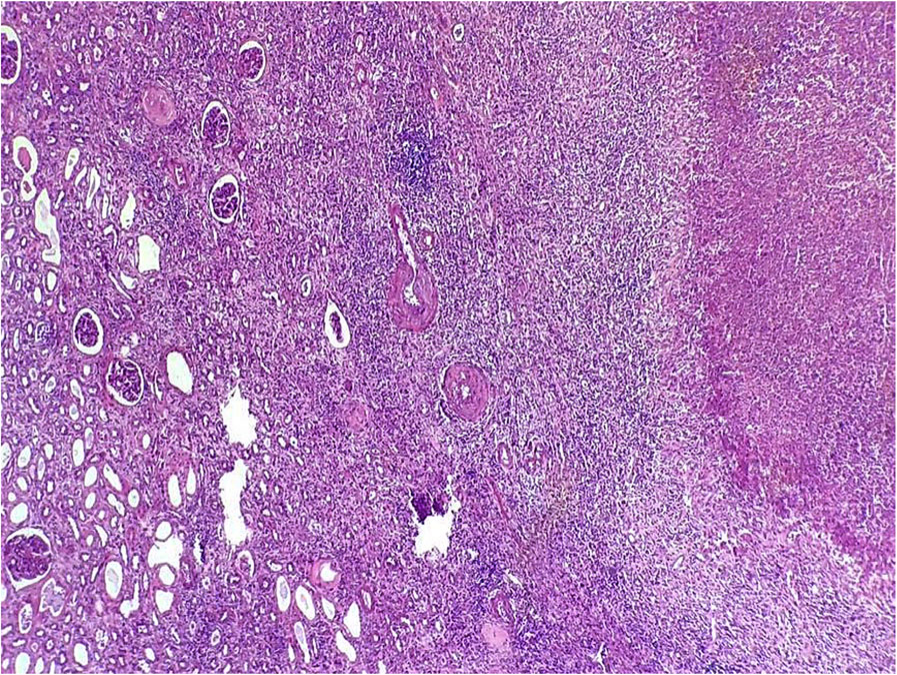
Pathology report revealed granulomatous inflammation with central necrosis of the kidney visualized with hematoxylin and eosin stain (original magnification × 40)

**Fig. 3:**
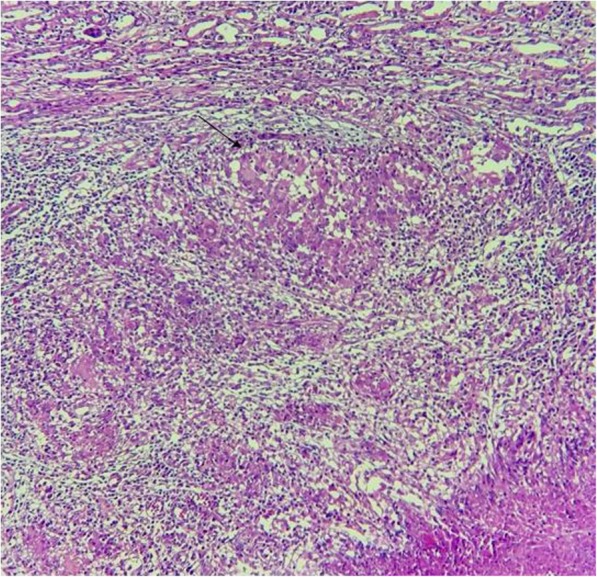
Granulomatous lesion including Langerhans giant cells visualized with hematoxylin and eosin stain (original magnification × 200)

The patient’s postoperative course was uneventful. Surprisingly, histopathological examination of the specimen revealed numerous confluent caseating granulomas with areas of dense inflammation extending into the perinephric fat, suggesting renal tuberculosis (Figs. 2 and 3). The patient had received bacille Calmette-Guérin vaccination as a child. A cutaneous tuberculin test was performed (12 mm), and ten samples of urine for mycobacterial culture and bronchoscopy with culture for Koch bacilli from the bronchoalveolar lavage were obtained. All mycobacterial culture results were negative. The result of a QuantiFERON-TB Gold test (Quest Diagnostics, Secaucus, NJ, USA) was positive. Treatment with antituberculosis drugs was started and continued for 6 months. She was in good health after 35 months of follow-up.

## Discussion

Urogenital tuberculosis is diagnosed in 1.1–1.5% of all tuberculosis cases and in 5–6% of cases of extrapulmonary tuberculosis [2]. This infection is usually a consequence of local reactivation following hematogenous dissemination of *Mycobacterium tuberculosis* to the renal cortex during primary pulmonary infection. The renal cortex is also frequently involved with miliary tuberculosis when multiple granulomas are present. The high oxygen tension of the renal cortex is favorable for renal localization [3]. The clinical presentation of urogenital tuberculosis consists of mostly nonspecific symptoms such as frequent urination, pyuria, dysuria, flank pain, fever, and weight loss [4]. Renal seeding following hematogenous spread from the primary site of infection is followed by formation of small inactive granulomas, which give rise to active tuberculosis after a long latent period, and therefore patients usually present in the second to fourth decades of their lives [4]. Intravenous pyelogram was traditionally the standard imaging approach, but computed tomography (CT) is now preferred [3]. The characteristic early finding is erosions of the renal calyx; the erosions subsequently progress to papillary necrosis, hydronephrosis, renal parenchymal cavitation, and dilated calyces. A thickened ureteric wall and structures characterize ureteric tuberculosis. Lesions are most common in the distal third of the ureter. Bladder tuberculosis may manifest as reduced bladder volume with wall thickening, ulceration, and filling defects resulting from granulomatous involvement. CT findings include focal caliectasis, hydronephrosis, calcifications, cortical thinning, and soft tissue masses [1]. Usually, an enhancing renal mass is caused by renal cell carcinoma, metastasis, lymphoma, or an abscess [1, 5]. Urogenital tuberculosis rarely manifests as pseudotumors, which otherwise are usually due to hypertrophied column of Bertin, renal dysmorphism, or an unusually shaped kidney [1, 6]. In rare cases, urogenital tuberculosis manifests as either single or multiple parenchymal nodules without urinary tract involvement. Patients with this form, known as the pseudotumoral type, present with variably sized but well-defined parenchymal nodules on cross-sectional images [1]. The lesion may simulate a renal hydatid cyst or a pseudotumoral xanthogranulomatous pyelonephritis. In extremely rare cases, however, genitourinary tuberculosis may present as well-defined parenchymal nodules of variable size, with sparing of the collecting system in what is known as pseudotumoral type. With the clinical and radiological findings suggestive of renal cell carcinoma, the patient consequently undergoes surgical removal of the involved kidney, whose histopathological examination unexpectedly establishes the diagnosis of tuberculosis [3]. The diagnosis is confirmed by growth of *Mycobacterium tuberculosis* in urine or tissue culture. The treatment of urogenital tuberculosis is similar to that of extrapulmonary tuberculosis at other sites. The initial regimen consists of four drugs (isoniazid, rifampin, pyrazinamide, and ethambutol) for 2 months, followed by two drugs (isoniazid, rifampin) for 4 months if the isolate is susceptible to first-line therapy.

## Conclusion

Pseudotumoral presentation of urogenital tuberculosis is very rare. Renal tuberculosis should be suspected when atypical renal masses are seen in patients from tuberculosis-endemic areas. Biopsy should be performed in cases of doubtful kidney tumors to provide an exact diagnosis. Treatment includes surgery followed by antitubercular therapy. An early diagnosis can save the kidney.
